# Clinical characteristics and prognosis of non-APAP drug-induced acute liver failure: a large multicenter cohort study

**DOI:** 10.1007/s12072-023-10541-w

**Published:** 2023-05-19

**Authors:** Lin Han, Ang Huang, Jinjun Chen, Guangju Teng, Ying Sun, Binxia Chang, Hong-Li Liu, Manman Xu, Xiaoqin Lan, Qingsheng Liang, Jun Zhao, Hui Tian, Songhai Chen, Yun Zhu, Huan Xie, Tong Dang, Jing Wang, Ning Li, Xiaoxia Wang, Yu Chen, Yong-Feng Yang, Dong Ji, Zhengsheng Zou

**Affiliations:** 1 Senior Department of Hepatology, The Fifth Medical Center of PLA General Hospital, Beijing, 100039 China; 2 State Key Laboratory of Organ Failure Research, Guangdong Provincial Key Laboratory of Viral Hepatitis Research, Department of Infectious Diseases and Hepatology Unit, Nanfang Hospital, Southern Medical University, Guangzhou, China; 3 Southeast University School of Medicine, No. 87 Dingjiaqiao Road, Gulou District, Nanjing, 210003 China; 4 The Second Hospital of Nanjing, Teaching Hospital of Southeast University, No. 1-1 Zhongfu Road, Gulou District, Nanjing, 210003 China; 5 Fourth Department of Liver Disease, Beijing Youan Hospital, Capital Medical University, No. 8, Xi Tou Tiao, Youanmenwai Street, Fengtai District, Beijing, 100069 China; 6 Beijing Municipal Key Laboratory of Liver Failure and Artificial Liver Treatment Research, No. 8, Xi Tou Tiao, Youanmenwai Street, Fengtai District, Beijing, 100069 China; 7 Inner Mongolia Institute of Digestive Diseases, The Second Affiliated Hospital of Baotou Medical College, Inner Mongolia University of Science and Technology, Baotou, China; 8 Department of Medical Risk Management, The Fifth Medical Center of PLA General Hospital, Beijing, China; 9 Department of Liver Diseases, The Second Hospital of Nanjing, Affiliated to Nanjing University of Traditional Chinese Medicine, No. 1-1 Zhongfu Road, Gulou District, Nanjing, 210003 China; 10 Peking University 302 Clinical Medical School, Beijing, 100039 China

**Keywords:** Acute liver failure, Drug-induced liver injury, Transplant-free survival, Hepatic encephalopathy

## Abstract

**Background:**

There is growing recognition of natural history, complications, and outcomes of patients who develop non-acetaminophen (APAP) drug-induced acute liver failure (ALF). To clarify high-risk factors and develop a nomogram model to predict transplant-free survival (TFS) in patients with non-APAP drug-induced ALF.

**Methods:**

Patients with non-APAP drug-induced ALF from 5 participating centers were retrospectively analyzed. The primary endpoint was 21-day TFS. Total sample size was 482 patients.

**Results:**

Regarding causative agents, the most common implicated drugs were herbal and dietary supplements (HDS) (57.0%). The hepatocellular type (*R* ≥ 5) was the main liver injury pattern (69.0%). International normalized ratio, hepatic encephalopathy grades, the use of vasopressor, *N*-acetylcysteine, or artificial liver support system were associated with TFS and incorporated to construct a nomogram model (drug-induced acute liver failure-5, DIALF-5). The AUROC of DIALF-5 for 7-day, 21-day, 60-day, and 90-day TFS in the internal cohort were 0.886, 0.915, 0.920, and 0.912, respectively. Moreover, the AUROC of DIALF-5 for 21-day TFS had the highest AUROC, which was significantly higher than 0.725 of MELD and 0.519 of KCC (*p* < 0.05), numerically higher than 0.905 of ALFSG-PI but without statistical difference (*p* > 0.05). These results were successfully validated in the external cohort (147 patients).

**Conclusions:**

Based on easily identifiable clinical data, the novel DIALF-5 model was developed to predict transplant-free survival in non-APAP drug-induced ALF, which was superior to KCC, MELD and had a similar prediction performance to ALFSG-PI but is more convenient, which can directly calculate TFS at multiple time points.

**Supplementary Information:**

The online version contains supplementary material available at 10.1007/s12072-023-10541-w.

## Introduction

Acute liver failure (ALF) is characterized by a rapid deterioration of liver function, resulting in altered mentation and coagulopathy in individuals without preexisting cirrhosis and with an illness of < 26 weeks’ duration [1, 2]. Its leading cause in the USA and European countries is drug-induced hepatotoxicity [3], which is stratified into intrinsic and idiosyncratic drug-induced liver injury (DILI). The best-known and widely used drug that can cause intrinsic DILI is acetaminophen (APAP) in North America [4] and the UK [5]. Non-APAP DILI, which ranks second among the overall causes of ALF in Western countries[3, 4], can be induced by prescription medications and Herbal and diet supplement (HDS) [6]. HDS are most commonly implicated in some Asian countries [7] and are increasingly implicated in Western countries as well [8, 9]. HDS products surpass pharmaceuticals in China accounting for 26.81% of DILI cases [7]. The incidence of APAP overdose (intrinsic DILI) is considerably lower or absent in most Asian countries [3]. Because of the higher annual incidence of non-APAP DILI in China [7], non-APAP drug-induced ALF is more common in Asian countries, especially in China, than in Western countries and is related to increased mortality.

Although only 10% of these patients with idiosyncratic DILI develop acute liver failure, up to 80% of these ALF patients die or require emergency liver transplantation (LT) due to the lack of effective treatment [3, 10–12]. In contrast to APAP injuries, non-APAP DILI reaching the threshold of acute liver failure presents higher bilirubin levels and worse overall outcomes, especially after any degree of hepatic encephalopathy (HE) has occurred [3, 13]. Transplant-free survival (TFS) in APAP-ALF has been improved in recent years, possibly due to administration of *N*-acetylcysteine (NAC) [6] and increased use of continuous renal replacement therapy [2]. Regrettably, this improved prognosis is limited among the wider array of non-APAP drug-induced ALF cases that are more difficult to diagnose and treat.

There is a paucity of data regarding non-APAP drug-induced ALF among adults in Asian countries, especially in China. There is growing recognition of natural history, complications, and outcomes of patients who develop non-APAP drug-induced ALF. Therefore, this study aimed to describe the clinical characteristics of non-APAP drug-induced ALF and develop an easy-to-use nomogram model to predict TFS at multiple time points from a large retrospective multicenter cohort study.

## Patients and methods

### Study design

The patients were screened from 5 participating hospitals in different regions of China. Clinical features of enrolled patients were summarized. The risk factors associated with TFS were identified by using univariable and multivariable Cox regression, and then incorporated into the nomogram to establish a prediction model. Afterward, the newly established model was validated using the data from the other 4 hospitals (external cohort). This study was performed according to the ethical guidelines of the 1975 Declaration of Helsinki, as revised in 1983. It was approved by the Ethics Committees of the Fifth Medical Center of Chinese PLA General Hospital (No. 2015–139-D). Written informed consent for data collection was waived due to the retrospective study design.

### Participants

The inclusion criteria were as follows: (1) age over 18 years old, (2) diagnosed with non-APAP DILI according to the American College of Gastroenterology (ACG) DILI Guidelines [14], and (3) evidence of ALF according to the enrollment criteria (see the operational definitions).

The exclusion criteria were as follows: (1) APAP toxicity etiology, (2) diagnosed with chronic DILI, (3) evidence of cirrhosis, and (4) incomplete data. No patients with severe acute liver injury were enrolled in this cohort study [15].

### Operational definitions

For this study, ALF was defined using the following criteria [1, 2, 16]: (1) evidence of coagulopathy with international normalized ratio (INR) ≥ 1.5, (2) HE of any grade (West Haven Criteria), (3) acute illness onset less than 26 weeks after the hepatic insult, and (4) an absence of preexisting cirrhosis. Follow-up began on the ALF diagnosis date and continued until death, liver transplantation, or 91 days after the index date.

DILI was confirmed if the patient had (1) ingested a drug, herbal, or dietary supplement prior to the DILI diagnosis; (2) Roussel Uclaf Causality Assessment Method (RUCAM) score ≥ 6 [17]; and (3) with no other etiology. A careful and detailed history of exposure to drugs was obtained from the patients (when possible) or their relatives.

In non-APAP drug-induced ALF, KCC is defined as either (1) INR > 6.5 (prothrombin time > 100 s) or (2) any 3 of the following variables: (i) age < 10 or > 40 years, (ii) etiology: non-A, non-B hepatitis, idiosyncratic drug reactions, (iii) duration of jaundice before the development of encephalopathy > 7 days, (iv) prothrombin time (PT) > 50 s (approx. INR > 3.5), and (v) serum bilirubin > 300 µmol/L [18]. The Acute Liver Failure Study Group prognostic index (ALFSG-PI) has been described as Logit SS = 2.67 – 0.95 (HE*) + 1.56 (Etiology*) – 1.25 (Vasopressor use*) − 0.70 (ln bilirubin) − 1.35 (ln INR). The logit SS can be transformed into the predicted probability of SS with the following formula: predicted SS = 1/(1 + e (− 1 * Logit SS)). (*For Light HE insert 0, for Deep HE insert 1; for Unfavorable Etiology insert 0, for Favorable Etiology insert 1; for absence of vasopressor use insert 0, for vasopressor use insert 1). ALFSG-PI has been applied previously to predict the TFS of patients with ALF based on easily identifiable hospital admission data [19]. The model for the end-stage liver disease (MELD) score was calculated as follows: [3.78 × ln(bilirubin in mg/dL) + 11.2 × ln(INR) + 9.57 × ln(creatinine in mg/dL) + 6.43] [20].

Acute kidney injury(AKI) is diagnosed by an increase in serum creatinine ≥ 0.3 mg/dL within 48 h or ≥ 50% increase in serum creatinine that is known or presumed to have occurred within the preceding 7 days [21, 22]. HE was defined as neuropsychiatric abnormalities during the course of liver disease, including involvement of the cognitive, affective/emotional, behavioral, and bioregulatory domains and graded by West Haven Criteria [23].

### Data collection and endpoints

The demographic, clinical, biochemical, and outcome data were retrieved from the medical electronic records. Data assessed in this study included baseline patient characteristics (age, sex, ethnicity, implicated drugs), biochemistry profiles (complete blood count, INR, albumin (ALB), serum alanine aminotransferase (ALT), aspartate aminotransferase (AST), total bilirubin (TBIL), alkaline phosphatase (ALP), ammonia, creatinine, alpha fetoprotein (AFP), total cholesterol (TC), triglyceride (TG)), complications (ascites, infection, AKI, HE), disease severity score (MELD, KCC for Non-APAP, ALFSG-PI), treatment (*N*-acetylcysteine use, glucocorticoid use, vasopressor use, artificial liver support system (ALSS) (mainly plasma exchange, PE)), and clinical outcomes (TFS).

### Statistical analysis

If the continuous variables were normally distributed, the data are presented as the mean ± standard deviation (mean ± SD). When they were not normally distributed, the median (interquartile range, IQR) was presented. Categorical data are expressed as numbers with percentages. The Mann–Whitney *U* test and Student’s *t* test were used to compare the nonparametric and parametric continuous variables, respectively. Categorical variables were analyzed by Chi-square tests or Fisher’s exact test. A two-tailed *p* < 0.05 was considered statistically significant. The Kaplan–Meier analysis and the log-rank test were used to compare the TFS in subgroups strafed by risk factors. Data were analyzed using R software, version 4.2.1 (R Foundation for Statistical Computing, Vienna, Austria; http://www.r-project.org/).

The independent risk factors associated with TFS in the internal cohort were identified by using univariable and multivariable Cox analyses, which were performed to estimate the hazard ratio (HR) and 95% confidence interval (CI) and then were incorporated to construct a nomogram, which was based on proportionally converting each multivariate regression coefficient to a 0- to 100-point scale, by using the replot package of *R*. The points are added across independent variables to derive total points and then convert to predicted probabilities of TFS at multiple time points.

The predictive performance of the nomogram was measured by time-dependent ROC created by using the timeROC package of *R*. Calibration curve was determined by bootstrap sampling to decrease the overfitting bias. The closer the calibration curve is to the diagonal line, the higher the prediction accuracy of the model. The *N*-time K-fold cross-validation was conducted to further evaluate the stability of predictive model by using the caret package of *R*. The model was also validated in the external cohort by using the above relevant methods.

## Results

### Clinical characteristics

**Fig. 1:**
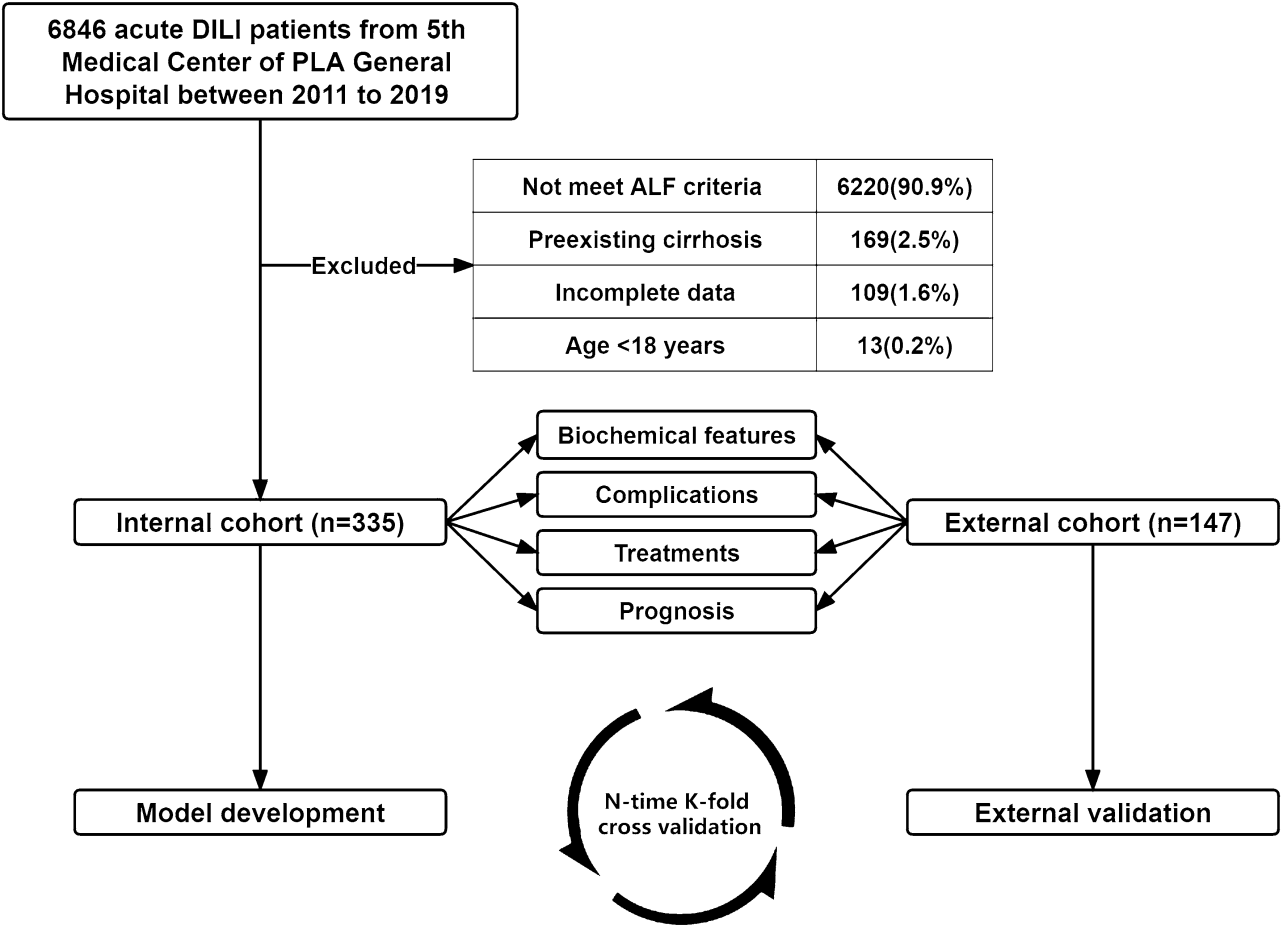
Flowchart of the study population. DILI, drug-induced liver injury; ALF, acute liver failure; APAP, acetaminophen

As shown in Fig. 1, a total of 6846 consecutive patients were screened. According to the inclusion and exclusion criteria, 6511 patients were excluded (6220 patients were ineligible according to the ALF criteria, 169 patients had preexisting cirrhosis, 109 patients had incomplete data, and 13 patients were younger than 18 years old). Finally, 335 patients with non-APAP drug-induced ALF were enrolled in the internal cohort between January 2011 and December 2019. The external cohort included 147 patients from the other 4 hospitals between October 2012 and April 2022. Total sample size was 482 patients.

The baseline demographic and laboratory characteristics of the 335 internal cohort patients are summarized in Table 1. The mean age was 46.1 years, and 237 patients (70.7%) were women. In terms of causative agents, 191 (57.0%) patients had taken HDS, 144 (43.0%) patients had taken drugs. The hepatocellular type (*R* ≥ 5) was the main liver injury pattern (69.0%). The main complication was ascites (68.1%), followed by infection (58.5%), high-grade (III–IV) HE (44.5%), AKI (27.2%). The clinical characteristics of the external cohort are similar to those of the internal cohort. (N-time K-fold “cross-validation” in above Figure 1)

**Table 1:** Baseline clinical features of patients with non-APAP drugs-induced acute liver failure

	Internal cohort				External cohort (*n* = 147)
	Total (*n* = 335)	Survival (*n* = 143)	Death/LT (*n* = 192)	*p* value	
Gender, female, n (%)	237 (70.7)	97 (67.8)	140 (72.9)	0.312	81 (55.1)
Age (years)	46.1 ± 14.9	45.1 ± 14.6	46.7 ± 15.2	0.324	48.8 ± 15.9
Ethnicity, Han, *n* (%)	318 (94.9)	139 (97.2)	179 (93.2)	0.101	145 (98.6)
*Implicated drugs, n (%)*				0.217	
Drugs	144 (43.0)	67 (46.9)	77 (40.1)		79 (53.7)
HDS	191 (57.0)	76 (53.1)	115 (59.9)		68 (46.3)
*R value, n (%)*				0.180	
Hepatocellular	231 (69.0)	95 (66.4)	136 (70.8)		88 (59.9)
Cholestatic	65 (19.4)	26 (18.2)	39 (20.3)		25 (17.0)
Mixed	39 (11.6)	22 (15.4)	17 (8.9)		34 (23.1)
*Baseline index*					
HGB, g/L (113–151)	119.0 (104.0, 134.0)	121.0 (111.0, 138.0)	117.5 (98.8, 132.3)	0.005	120.0 (106.0, 135.0)
PLT, 10^9^/L (101–320)	143.0 (88.0, 208.0)	165.0 (109.5, 227.5)	125.0 (72.8, 196.0)	< 0.001	131.0 (92.0, 186.0)
INR, (0.8–1.2)	1.9 (1.7, 2.6)	1.8 (1.6, 2.0)	2.3 (1.8, 3.3)	< 0.001	2.0 (1.6, 3.0)
Albumin, g/L (35–55)	29.0 (26.0, 32.0)	30.0 (27.0, 33.0)	28.0 (25.0, 32.0)	0.001	31.0 (28.0, 34.0)
ALT, U/L (5–35)	393.0 (157.0, 868.0)	403.5 (157.5, 905.8)	388.0 (155.0, 858.0)	0.970	375.0 (148.5, 889.0)
AST, U/L (8–40)	384.0 (176.0, 836.0)	315.5 (147.3, 704.8)	460.0 (204.0, 909.0)	0.010	373.0 (159.8, 656.0)
TBIL, µmol/L (3.4–20.5)	321.2 (233.6, 414.2)	286.4 (220.7, 400.0)	339.0 (247.8, 436.6)	0.009	320.8 (212.4, 435.4)
ALP, U/L (40–150)	160.0 (132.0, 200.0)	156.0 (136.0, 196.0)	160.5 (129.0, 202.5)	0.806	136.1 (112.0, 181.0)
CR, µmol/L (53–97)	77.0 (63.0, 100.8)	74.0 (61.8, 85.3)	80.0 (65.5, 140.3)	0.002	55.0 (44.0, 68.5)
AMMO, µmol/L (0–30)	89.0 (62.1, 161.0)	64.8 (52.7, 84.2)	143.0 (81.3, 197.9)	< 0.001	100.5 (67.5, 143.8)
AFP, ng/mL (0–10.0)	57.0 (11.3, 193.9)	78.3 (16.0, 262.6)	45.0 (8.7, 153.4)	0.018	49.9 (9.3, 200.8)
TC, mmol/L (2.8–5.2)	1.8 (1.3, 2.5)	2.0 (1.5, 2.6)	1.7 (1.1, 2.4)	0.002	2.8 (2.4, 3.6)
TG, mmol/L (0.56–1.7)	1.4 (0.9, 2.1)	1.3 (0.9, 2.2)	1.4 (0.9, 2.0)	0.663	1.1 (0.7, 1.7)
*Complication (n, %)*					
Ascites	228 (68.1)	88 (61.5)	140 (72.9)	0.027	71 (48.3)
Infection	196 (58.5)	53 (37.1)	143 (74.5)	< 0.001	97 (66.0)
Acute kidney injury	91 (27.2)	12 (8.4)	79 (41.1)	< 0.001	21 (14.3)
HE grade				< 0.001	
I	151 (45.1)	115 (80.4)	36 (18.8)		98(66.7)
II	35 (10.4)	19 (13.3)	16 (8.3)		26(17.7)
III–IV	149 (44.5)	9 (6.3)	140 (72.9)		23(15.6)
*Disease severity score*					
MELD	24.6 (21.1, 29.6)	22.5 (19.3, 25.0)	26.9 (22.7, 33.1)	< 0.001	21.7 (17.3, 26.0)
KCC-non-APAP (*n*, %)	237 (70.7)	85 (59.4)	152 (79.2)	< 0.001	121(82.3)
ALFSG-PI	0.3 (0.1, 0.5)	0.5 (0.4, 0.5)	0.1 (0.1, 0.3)	< 0.001	0.4 (0.2, 0.5)
*Treatment (n, %)*					
Vasopressor	86 (25.7)	2 (1.4)	84 (43.8)	< 0.001	12 (8.2)
*N*-acetylcysteine	82 (24.5)	47 (32.9)	35 (18.2)	0.002	30 (20.4)
Glucocorticoid	43 (12.8)	22 (15.4)	21 (10.9)	0.229	36 (24.5)
ALSS	108 (32.2)	52 (36.4)	56 (29.2)	0.163	36 (24.5)

We compared the clinical and biochemical features of enrolled patients in internal cohort stratified by outcome (TFS or death/LT) (Table 1). There were no statistically significant differences for age, ethnicity, sex ratio, implicated drugs, R value, ALT, ALP, or TG. Patients who died or underwent LT usually had lower hemoglobin (HGB), platelet (PLT), TC, and AFP levels, worse indices of ALB, AST, INR, TBIL, ammonia, serum creatinine, disease severity scores (MELD, KCC for non-APAP, and ALFSG-PI) and more clinically detectable complications (ascites, infection, AKI, and high-grade (III–IV) HE than TFS patients (*p* < 0.05 for all).

### Drugs implicated in non-APAP drug-induced ALF

As shown in Supplementary Fig. 1, the implicated drugs were categorized according to their class and main clinical indication. The leading cause of non-APAP drugs-induced ALF was HDS (57.01%), followed by drugs (42.99%) (Supplementary Fig. 1A). The common implicated drugs were nonsteroidal anti-inflammatory drugs (NSAIDs, excluding APAP) (16.42%), anti-tuberculosis drugs (6.57%), and anti-infectious agents (5.37%) (Supplementary Fig. 1B). Supplementary Fig. 1C shows the distribution of primary diseases that HDS was used to treat the patients before the onset of ALF.

### TFS in subgroups

**Fig. 2:**
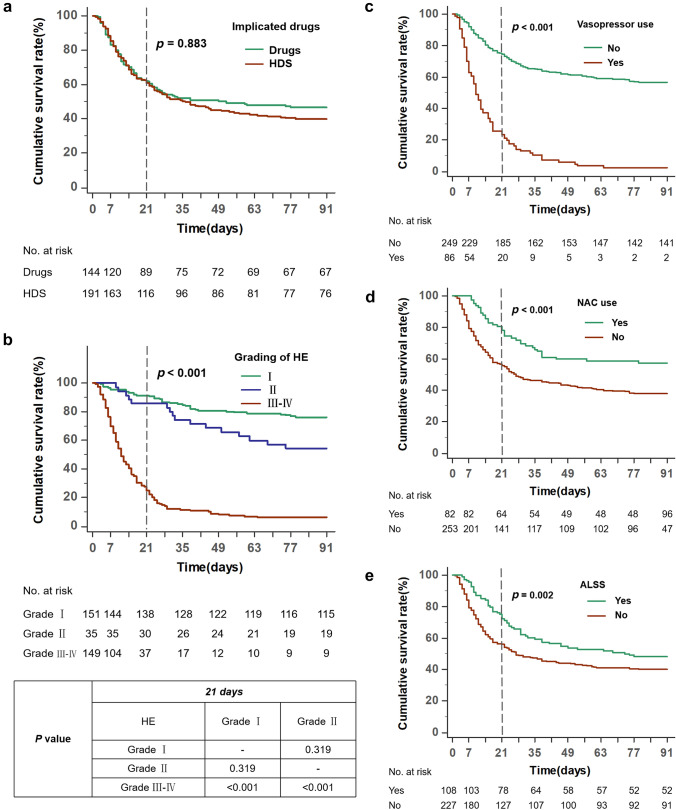
Kaplan–Meier analysis of transplant-free survival **A** by implicated drugs, **B** by grading of HE, **C** by vasopressor use, **D** by NAC use, **E** by ALSS use. Cumulative transplant-free survival across groups was compared using the log-rank test. 0 in *X*-axis indicates the time when ALF was diagnosed. HDS, herbal and dietary supplements; HE, hepatic encephalopathy; NAC, *N*-acetylcysteine; ALSS, artificial liver support system

The overall median follow-up time was 38.0 days (IQR: 11.0–91.0). The TFS rates in the drugs group, and the HDS group at 21-day were 61.8%, and 60.7%, respectively (*p* = 0.883, Fig. 2A). The TFS rates in HE grade I, II and III–IV group at 21-day were 91.4%, 85.7%, and 24.8%, respectively (*p* < 0.001, Fig. 2B). The TFS rate at 21-day was 74.3% in subgroup without vasopressor use, which was higher than that of the with vasopressor use (23.3%, *p* < 0.001, Fig. 2C). The TFS rate at 21-day was 78.0% in subgroup with NAC use, which was higher than that of the without NAC use (55.7%, *p* < 0.001, Fig. 2D). The TFS rate at 21-day was 72.2% in subgroup with ALSS, which was higher than that of the without ALSS (55.9%, *p* = 0.002, Fig. 2E). In the internal cohort, 37 patients underwent liver transplantation and 155 patients died during the 90-day follow-up period. In the external cohort, 16 patients underwent liver transplantation and 25 patients died during the 90-day follow-up period.

### Novel prediction nomogram development

**Fig. 3:**
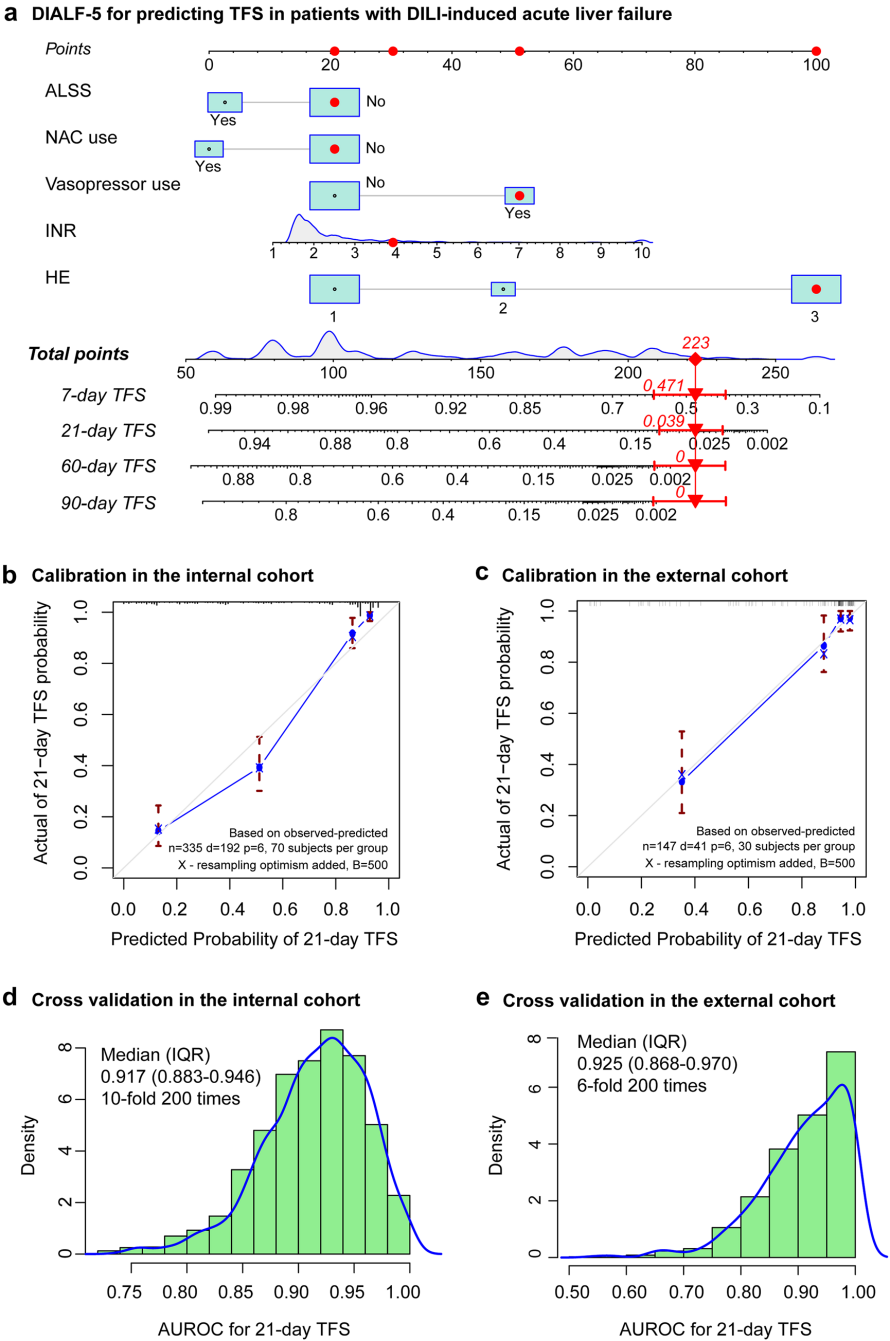
Discrimination and calibration of the non-APAP DIALF-5 model to predict risk of prognosis. **A** DIALF-5 model, **B** the calibration curve for 21-TFS in the internal cohort, **C** the calibration curve for 21-TFS in the external cohort, **D** the distribution of AUROC for 21-day TFS in the internal cohort, **E** the distribution of AUROC for 21-day TFS in the external cohort. DIALF, drugs-induced acute liver failure; TFS, transplant-free survival; ALSS, artificial liver support system; NAC, *N*-acetylcysteine; INR, international normalized ratio; HE, hepatic encephalopathy; AUROC, area under the receiver operating curve

Through univariable and multivariable Cox regression analyses, we selected statistically significant variables (Table 2) including HE (grade II, HR = 2.221; 95% CI 1.184–4.167, *p* = 0.013; grade III or IV, HR = 8.905, 95% CI 5.429–14.607, *p* < 0.001), vasopressor use (HR = 2.077, 95% CI 1.380–3.126, *p* < 0.001), NAC use (HR = 0.553, 95% CI 0.371–0.823, *p* = 0.003), ALSS (HR = 0.553, 95% CI 0.387–0.790, *p* = 0.001), and INR (HR = 1.193, 95% CI 1.098–1.295, *p* < 0.001) as candidates to construct the novel prediction model and named as the DIALF-5 (drug-induced acute liver failure-5) (Fig. 3A), which showed the well calibration by 500 times bootstrap sampling (Fig. 3B, C) and good accuracy in predicting the probability of 21-day TFS, with a median AUROC of 0.917 (IQR: 0.883–0.946) in the internal cohort and 0.925 (IQR: 0.868–0.970) in the external validation cohort by cross-validation (Fig. 3D, E).

**Table 2:** Univariable and multivariable Cox regression analyses for prognosis of non-APAP drugs-induced acute liver failure cases in the internal cohort

Parameters	Univariable		Multivariable	
	OR (95% CI)	*P* value	OR (95% CI)	*P* value
Female	1.130 (0.821, 1.550)	0.458		
Age (years)	1.000 (0.994, 1.010)	0.508		
Ethnicity (Han)	0.623 (0.354, 1.090)	0.099		
Implicated drugs (HDS)	1.150 (0.858, 1.530)	0.357		
*R value*	–	–		
Hepatocellular	–	–		
Cholestatic	0.904 (0.633, 1.291)	0.580		
Mixed	0.609 (0.368, 1.009)	0.054		
*Baseline index*				
HGB, g/L	0.989 (0.983, 0.995)	<0.001		
PLT, 10^9^/L	0.997 (0.995, 0.998)	< 0.001		
INR	1.320 (1.240, 1.410)	< 0.001	1.193 (1.098, 1.295)	< 0.001
Albumin, g/L	0.930 (0.899, 0.961)	< 0.001		
ALT,U/L	1.000 (1.000, 1.000)	0.762		
AST, U/L	1.000 (1.000, 1.000)	0.108		
TBIL, umol/L	1.000 (1.000, 1.000)	0.166		
ALP, U/L	1.000 (0.999, 1.000)	0.501		
CR, µmol/L	1.000 (1.000, 1.000)	0.003		
AMMO, µmol/L	1.010 (1.010, 1.010)	< 0.001		
AFP, ng/mL	1.000 (1.000, 1.000)	0.550		
TC, mmol/L	0.918 (0.818, 1.030)	0.148		
TG, mmol/L	0.978 (0.833, 1.150)	0.784		
*Complication*				
Ascites	1.480 (1.080, 2.040)	0.016		
Infection	2.800 (2.020, 3.890)	< 0.001		
Acute kidney injury	2.820 (2.110, 3.780)	< 0.001		
*HE grade*	–	–	–	–
I	–	–	–	–
II	2.080 (1.150, 3.740)	0.015	2.221 (1.184, 4.167)	0.013
III–IV	11.700 (8.000, 17.300)	< 0.001	8.905 (5.429, 14.607)	< 0.001
*Treatment*				
Vasopressor	5.480 (4.050, 7.420)	< 0.001	2.077 (1.380, 3.126)	< 0.001
*N*-acetylcysteine	0.529 (0.367, 0.764)	0.001	0.553 (0.371, 0.823)	0.003
Glucocorticoid	0.684 (0.435, 1.077)	0.101		
ALSS	0.707 (0.518, 0.966)	0.029	0.553 (0.387, 0.790)	0.001

HDS, herbal and dietary supplements; HGB, hemoglobin; PLT, platelet; INR, international normalized ratio; ALT, alanine aminotransferase; AST, aspartate aminotransferase; TBIL, total bilirubin; ALP, alkaline phosphatase; CR, creatinine; AMMO, ammonia; AFP, alpha fetoprotein; TC,total cholesterol; TG, triglyceride; HE, hepatic encephalopathy; ALSS, artificial liver support system

### Comparison of prognostic models in non-APAP drug-induced ALF

**Fig. 4:**
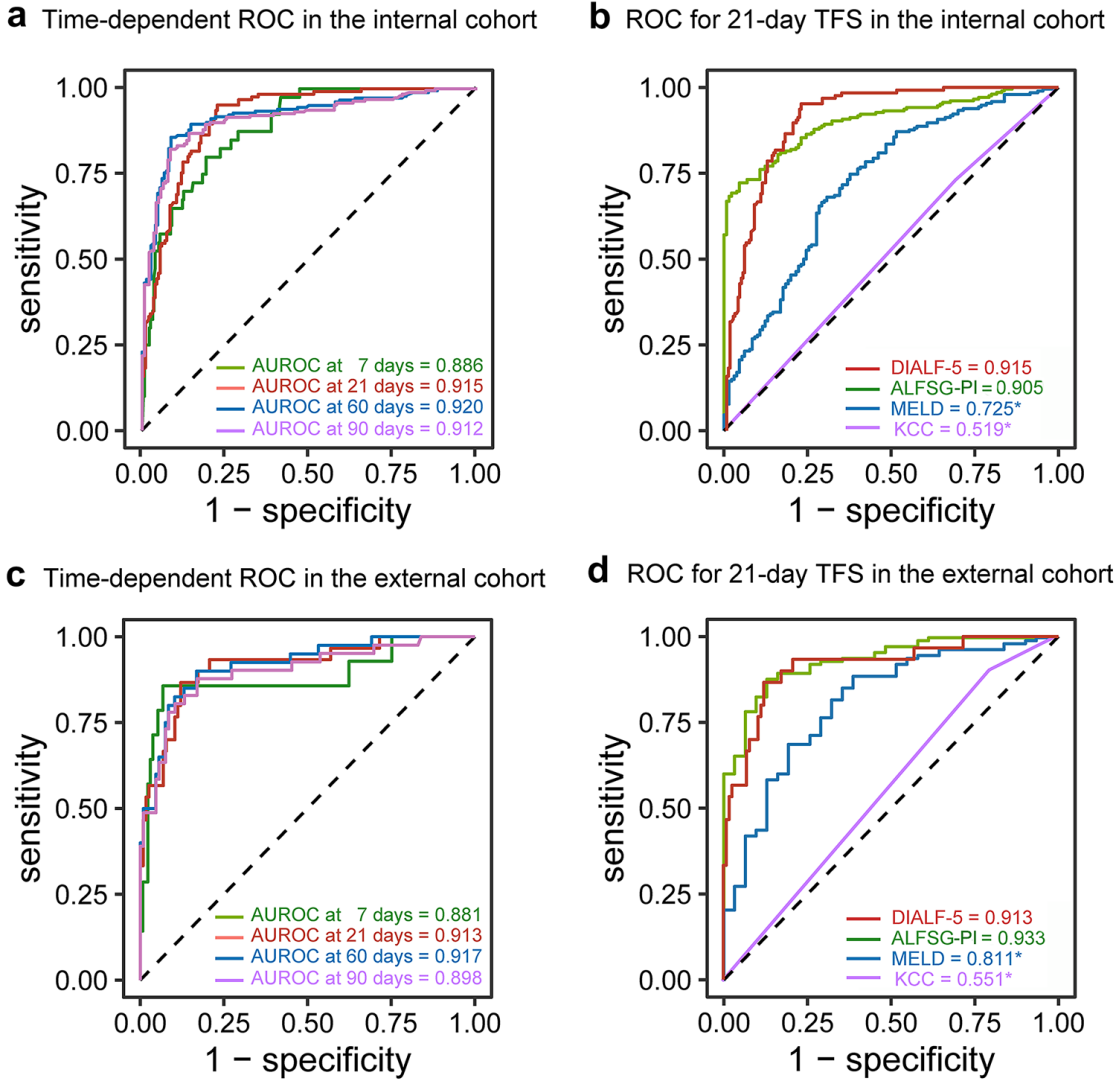
Prediction performance of DIALF-5 model. **A** time-dependent ROC for multiple time points TFS in the internal cohort, **B** ROC comparison for 21-day TFS in the internal cohort, **C** time-dependent ROC for multiple time points TFS in the external cohort, **D** ROC comparison for 21-day TFS in the external cohort. *Statistical significance compared with DIALF-5 model (*p* < 0.05). ALFSG-PI, Acute Liver Failure Study Group Prognostic Index; MELD, model of end stage liver disease; KCC: King’s College Criteria for Non-APAP; AUROC, area under the receiver operating curve

AUROC of DIALF-5 for 7-day, 21-day, 60-day, and 90-day TFS in the internal cohort were 0.886, 0.915, 0.920, and 0.912, respectively (Fig. 4A). The AUROC of DIALF-5 for 21-day TFS had the highest AUROC of 0.915, which was significantly higher than 0.725 of MELD and 0.519 of KCC (*p* < 0.05), numerically higher than 0.905 of ALFSG-PI but without statistical difference (*p* > 0.05, Fig. 4B). The external validation cohort had the similar results (Fig. 4C, D).

## Discussion

By using a large multicenter cohort study, we have summarized the clinical characteristics and prognosis of patients with non-APAP drug-induced ALF. According to our multivariate analyses, HDS were the main drugs implicated in non-APAP drug-induced ALF. Multivariable analyses showed that INR, vasopressor use, high-grade (III–IV) HE, the use of NAC, and ALSS (e.g., plasma exchange, PE) had independent impacts on TFS in patients with non-APAP drug-induced ALF. A nomogram model (DIALF-5) based on these 5 factors was developed from 335 cases, validated in an external cohort of 147 patients.

As reported in Western studies, non-APAP drug-induced ALF was most frequently associated with the use of antibiotics (including anti-tuberculous (TB) medications) [3, 13, 24, 25], which is different from a previous report that most cases of ALF were induced by traditional Chinese herbs in Asian countries [26]. However, in the past 25 years, the number of people waiting for liver transplantation has increased eight folds due to HDS-related liver failure in the United States [12]. Furthermore, liver failure caused by HDS is more commonly related to Asians in USA [27]. HDS are widely used to treat miscellaneous diseases in China because a significant proportion of Chinese individuals prefer to use HDS based on the mistaken belief that these drugs have few or no side effects. However, there is no standard classification of HDS, which include vitamins, minerals, elements, herbal and botanical remedies, multi-ingredient compounds, and non-prescription anabolic steroids [6, 27]. Therefore, when determining the etiology of ALF, physicians should pay more attention to the patient's past HDS treatment/using history.

Although the prognosis of ALF varies greatly with the underlying etiology, INR has been identified as one of the important prognostic variables among patients with ALF in many studies [11, 28]. In terms of outcomes, we showed that high-grade (III–IV) HE was significantly associated with worse TFS (*p* < 0.001). The occurrence of cerebral edema and intracranial hypertension, which have long been recognized as the most serious complications of ALF, is related to the severity of HE in ALF [1, 11].

In patients with acute liver failure, vasopressor (e.g., norepinephrine) use has been suggested by consensus as the preferred intervention for persistent hypotension (mean arterial pressure < 60 mmHg) despite volume repletion should prompt. Unfortunately, due to the continuous vasoconstriction of microcirculation after vasopressor use, terminal organ dysfunction, tissue hypoxia, and lactic acidosis may continue to occur. In our study, we also observed that vasopressor use was associated with worse 21-day TFS.

There is no certain or specific treatment for idiosyncratic drug-induced ALF. *N*-acetylcysteine (NAC) is an effective treatment for APAP overdoses (intrinsic DILI), but its role in non-APAP drug-induced ALF is controversial. NAC was shown to improve TFS in adult patients with early-stage non-APAP drug-induced ALF [29]. Similar to previous reports [30–33], the present study showed that early administration of NAC might be effective in treating adult patients with ALF due to idiosyncratic DILI.

Although ALSS might transiently improve biochemical indices of liver function, none has been shown to reliably bridge patients with ALF to transplantation or the recovery of liver function, mitigating the need for transplantation [3, 11]. In our study, we observed that plasma exchange was associated with improved 21-day TFS, similar to previous report [34]. The future of plasma exchange or other ALSS in the management of ALF remains unclear, and further study is required.

Accurate prognostic indices for ALF have been a focus of many investigations. Therefore, we analyzed prognostic scoring systems, including King’s College Criteria (KCC) for non-APAP, MELD score, the ALFSG-PI, and DIALF-5. In our study, of greater importance and practical value, DIALF-5 was more accurate than KCC for non-APAP and MELD in predicting TFS. Additionally, DIALF-5 had a similar prediction performance to ALFSG-PI but is more convenient, which can directly calculate TFS at multiple time points without using complex logarithmic formula, facilitating the estimation of individualized and stratified outcomes. A previous study showed that the ALFSG-PI was proposed to identify 21-day TFS in patients with ALF of all causes in Western countries [19]. At present, our findings indicate that the DIALF-5 is highly predictive of short- and long-term outcomes in non-APAP drug-induced ALF.

The strengths of the present study are as follows: (1) This was a multicenter cohort study for non-APAP drug-induced ALF with a large sample size (nearly 500 patients) from different regions of China, making it a strong representative and deducing estimates with narrow CIs; (2) The targeting patients of the present study were with non-APAP drug-induced ALF, which is common in Asian countries and is related to increased morbidity; (3) we conducted the study using advanced statistical methods (*N*-time K-fold cross-validation and 500-time bootstrap validation), providing more robust results.

This study has some limitations. First, unlike national registry data, which can be used as a surrogate for population-based studies, this was a retrospective study, but it is representative of large samples (nearly 500 patients) with non-APAP drug-induced ALF from multicenter. Second, a prospective study is required to confirm the conclusions of this study. Despite the two limitations, this study represents the most recent and largest cohort (nearly 500 patients) evaluating their clinical characteristics and short- and long-term outcome trends.

In conclusion, with the increasing popularity of HDS in China, even in other countries in Asia, the combination of drugs and HDS were found to be the major causes of non-APAP drug-induced ALF. Using data derived from the most recent and largest cohort in China, INR, HE grades, the use of vasopressor, NAC, or ALSS was found to be associated with TFS, and incorporated to construct a nomogram model (DIALF-5), which uses simple, readily available clinical and laboratory variables to provide an easy-to-use approach in predicting TFS at multiple time points in non-APAP drug-induced ALF.

### Supplementary Information

Below is the link to the electronic supplementary material.

Supplementary file1 (PDF 1455 KB)

## Data Availability

All data relevant to the study are included in the article or uploaded as supplementary information. Further inquiries can be directed to the corresponding authors.

## Abbreviations

AFP: Alpha fetoprotein
AKI: Acute kidney injury
ALB: Serum albumin
ALF: Acute liver failure
ALFSG: Acute liver failure study group
ALFSG-PI: ALFSG-prognostic index
ALSS: Artificial liver support system
ALT: Alanine aminotransferase
ALP: Alkaline phosphatase
AMMO: Ammonia
APAP: Acetaminophen
AST: Aspartate aminotransferase
AUROC: Area under the receiver operating characteristic curve
CR: Serum creatinine
DIALF: Drug-induced acute liver failure
DILI: Drug-induced liver injury
HDS: Herbal and dietary supplements
HE: Hepatic encephalopathy
HGB: Hemoglobin
INR: International normalized ratio
KCC: King’s College Criteria
MELD: Model for end-stage liver disease
LT: Liver transplantation
NAC: *N*-Acetylcysteine
NSAIDs: Nonsteroidal anti-inflammatory drugs
PLT: Platelet
RUCAM: Roussel Uclaf Causality Assessment Method
ULN: Upper limit of normal
TBIL: Total bilirubin
TC: Total cholesterol
TFS: Transplant-free survival
TG: Triglyceride
