# Supplementary material for: Clinical characteristics and prognosis of non-APAP drug-induced acute liver failure: a large multicenter cohort study
Source: Hepatol Int. 2023 May 19;18(1):225–37. doi: 10.1007/s12072-023-10541-w (PMC10858105; doi:10.1007/s12072-023-10541-w)
Supplement: Supplementary file 1 — Supplementary file1 (PDF 1455 KB) [file 12072_2023_10541_MOESM1_ESM.pdf]

Supplementary Figure 1 Implicated drugs in this study.

† excluding anti-tuberculosis drugs ‡ excluding tuberculosis

HDS, herbal and dietary supplements; APAP, acetaminophen.

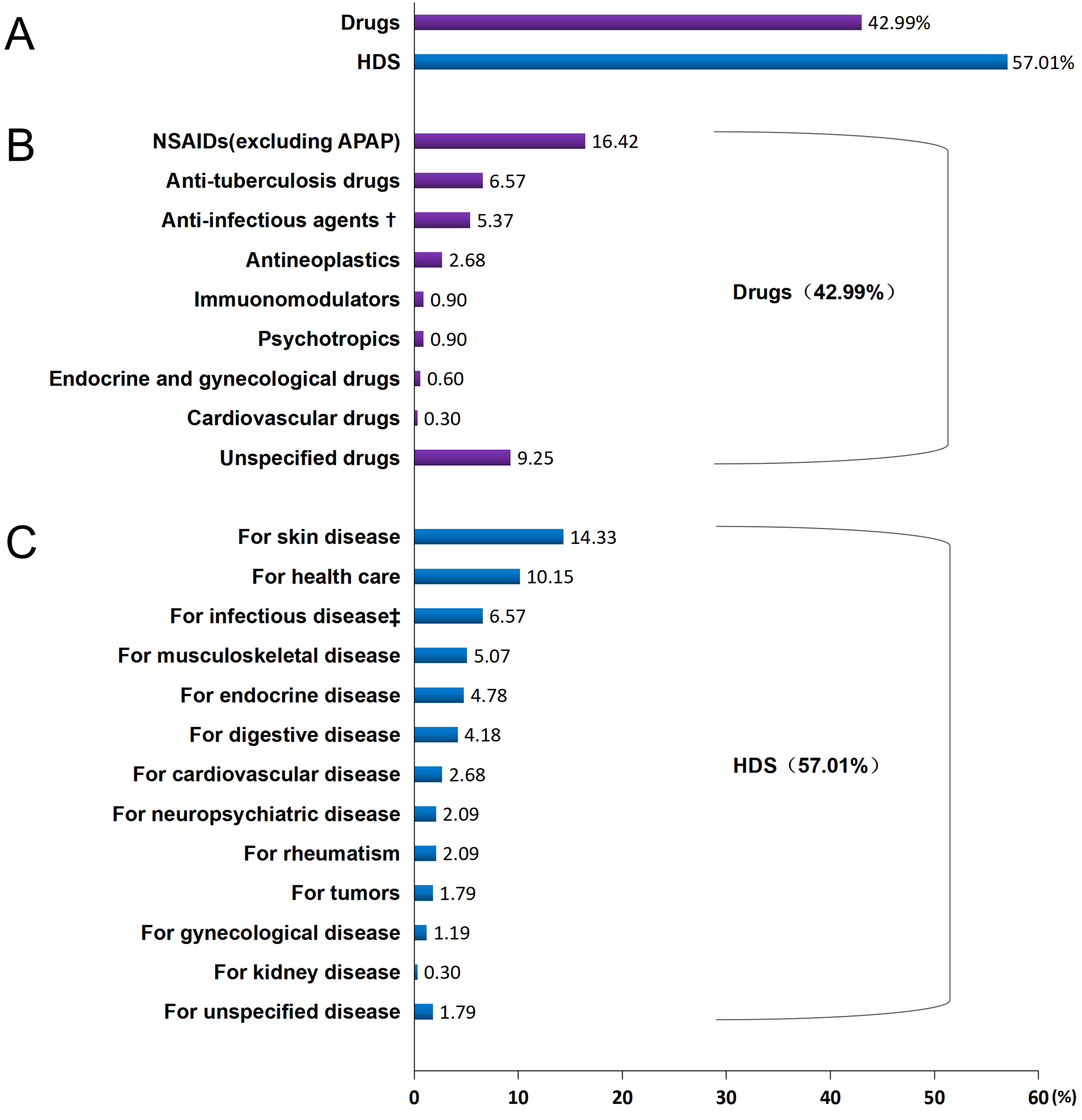
A
Drugs
42.99%
HDS
57.01%
B
NSAIDs(excluding APAP)
16.42
Anti-tuberculosis drugs
6.57
Anti-infectious agents †
5.37
Antineoplastics
2.68
Immuonomodulators
0.90
Psychotropics
0.90
Endocrine and gynecological drugs
0.60
Cardiovascular drugs
0.30
Unspecified drugs
9.25
Drugs（42.99%）
C
For skin disease
14.33
For health care
10.15
For infectious disease‡
6.57
For musculoskeletal disease
5.07
For endocrine disease
4.78
For digestive disease
4.18
For cardiovascular disease
2.68
For neuropsychiatric disease
2.09
For rheumatism
2.09
For tumors
1.79
For gynecological disease
1.19
For kidney disease
0.30
For unspecified disease
1.79
HDS（57.01%）
0
10
20
30
40
50
60
(%)
